# Wintering Together: Do Migrants Impact Residents? A Literature Review

**DOI:** 10.1002/ece3.70868

**Published:** 2025-02-16

**Authors:** Kimberly C. Navarro‐Velez, André A. Dhondt

**Affiliations:** ^1^ Department of Natural Resources and the Environment Cornell University Ithaca New York USA; ^2^ Cornell Lab of Ornithology Ithaca New York USA; ^3^ Department of Ecology and Evolutionary Biology Cornell University Ithaca New York USA

**Keywords:** agonistic behavior in sympatric species, exploitation competition in birds, foraging niche similarity, migrant‐resident bird interactions, overwintering dynamics, resource partitioning in tropical avifauna

## Abstract

Year after year, billions of long‐distance migratory birds join year‐round resident tropical birds during the overwintering period (dry season in the northern tropics) marked by harsh environmental conditions. The overlapping habitat use and apparent resource overlap during this time suggest potential interspecific competition between residents and migrants. Previous studies have explored the effects of such competition on migrants, but the impact on residents has been neglected. This review critically evaluates and summarizes the findings in the literature about interactions between migratory and resident birds in tropical ecosystems, using the necessary and sufficient conditions recommended by Dhondt to assess interspecific competition. This review reveals that interspecific territoriality, consistent aggression (interference competition), and alterations in foraging behavior and microhabitat impacted resident birds. High dietary overlap observed in some species pairs suggests potential exploitation competition, although our understanding of year‐round residents' diets and the full scope of these interactions remains limited. The complexity of these relationships underscores the need for comprehensive research to disentangle seasonal effects from competitive pressures and to assess impacts on resident fitness, distribution, and abundance. Inconsistent methodologies and inadequate spatio‐temporal scales have hindered a clear understanding of this phenomenon. We emphasize the importance of long‐term studies, including observations before and after migrant arrival at wintering grounds, to detect shifts in resource availability and foraging niches. Given climate change's influence on migration patterns and food resources, elucidating these biotic interactions is crucial for developing effective conservation strategies for resident tropical avifauna. Particularly, as many resident birds exhibit high levels of endemism, higher sedentarism, and specialized foraging habits, they may be more vulnerable to ecological changes than migrants, who are more flexible, generally occupying broader foraging niches. This review highlights critical knowledge gaps and proposes directions for future research to enhance our understanding of migrant‐resident bird dynamics in tropical ecosystems.

## Graphical abstract

This review examines the overlooked impacts of interspecific competition on resident tropical birds during the overwintering period, highlighting the need for a deeper understanding of their interactions with migratory species. By evaluating the literature through the lens of competition theory, we identify critical gaps in knowledge and suggest that long‐term studies are vital for assessing the ecological consequences of these dynamics.

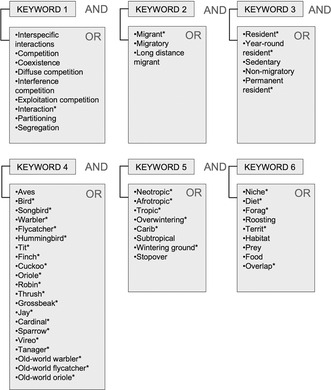

## RESUMEN

Año tras año, billones de aves migratorias de larga distancia se unen a aves tropicales residentes durante el periodo de invernada (estación seca en los trópicos del norte), marcado por duras condiciones ambientales. El uso compartido de hábitats y recursos sugiere una posible competencia interespecífica entre las aves residentes y migratorias. Estudios previos han explorado los efectos de dicha competencia en las aves migratorias, pero el impacto en las aves residentes ha sido pasado por alto. Esta revisión evalúa y resume críticamente los hallazgos en la literatura sobre las interacciones en ecosistemas tropicales, utilizando las condiciones necesarias y suficientes recomendadas por Dhondt (2012) para evaluar la competencia interspecifica. Se revela que la territorialidad interspecifica, la agresión consistente (competencia por interferencia) y las alteraciones en el comportamiento de forrajeo impactan las aves residentes. La alta similitud dietaria en algunas especies sugiere competencia por explotación, aunque la comprensión de las dietas de los residentes y el alcance de estas interacciones es limitada. La complejidad de estas relaciones subraya la necesidad de investigaciones exhaustivas que permitan diferenciar los efectos estacionales de las presiones competitivas y evaluar los impactos en la condición física, la distribución y la abundancia de las aves residentes. Metodologías inconsistentes y escalas espaciotemporales inadecuadas han obstaculizado una comprensión clara de este fenómeno. Se enfatiza la importancia de estudios a largo plazo que incluyan observaciones antes y después de la llegada de las aves migratorias a las zonas de invernada, para detectar cambios en la disponibilidad de recursos y nichos de forrajeo. Dada la influencia del cambio climático en la migración y los recursos alimenticios, esclarecer estas interacciones es crucial para desarrollar estrategias de conservación efectivas. En particular, las aves residentes, con altos niveles de endemismo y hábitos especializados, podrían ser más vulnerables a los cambios ecológicos en comparación con las aves migratorias, que son más flexibles y suelen ocupar nichos de forrajeo más amplios. Esta revisión identifica brechas en el conocimiento y propone directrices para futuras investigaciones que mejoren nuestra comprensión de las dinámicas entre aves migratorias y residentes en los ecosistemas tropicales.

## 1 Introduction

Every year, year‐round resident tropical birds (referred to as “residents”) are joined by billions of long‐distance migrants (referred to as “migrants”) who spend several months in the tropics (Hahn, Bauer, and Liechti 2009; Salewski and Jones 2006; Van Doren and Horton 2018). This seasonal stay occurs in the dry season, at the end of the main reproductive peak of most residents (Hau, Perfito, and Moore 2008) a period characterized by reduced food availability and harsher environmental conditions (Cooper et al. 2015; Poulin and Lefebvre 1996; Rabøl 1987; Waide 1981). Given the overlapping distribution and habitat use of many resident and migratory bird species during the food‐limited dry season, one might expect that individuals of species whose ecological niches overlap could experience interspecific competition (Dhondt 2012; Powell et al. 2021; Kent et al. 2022). Previous studies have concentrated on interspecific competition among migrants, on the impact of residents on migrants (Kent and Sherry 2020; Leisler, Heine, and Siebenrock 1983; Mostafa et al. 2021), and how the effects of competition carry over to the migrants' survival and reproductive fitness when they return to their breeding grounds (Cooper et al. 2015; Marra et al. 2015; Reudink et al. 2009; Rosenberg et al. 2019; Powell et al. 2021; Norris et al. 2004). However, the extent to which these interactions could influence the survival and reproductive fitness of the residents has mostly been ignored. Just a few studies have investigated the possibility of migrants impacting resident birds' foraging ecology (Chipley 1976; Rabøl 1987; O'Donnell, Kumar, and Logan 2014).

Here, we present a literature review summarizing the evidence supporting the hypothesis that long‐distance migrants impact residents in the tropics through direct and indirect competitive interactions. Understanding the possible effects of migrants on residents is critical for developing conservation strategies, especially as climate change impacts food abundance (Cooper et al. 2015; Poulin and Lefebvre 1996; Waide 1981), which could enhance competition. Additionally, as interspecific interactions are considered a key factor driving the evolution of migratory behavior in birds (Salewski and Bruderer 2007), research on interactions between migratory and resident species provides valuable insights into the ecological conditions that may have led to the emergence of migration (Guaraldo, Kelly, and Marini 2019). Therefore, this review addresses the following questions: is there any evidence that migrants impact resident birds? If so, is this a general phenomenon, and what are the identifiable patterns or mechanisms?

## 2 Methods

### 2.1 Search Protocol

**FIGURE 1:**
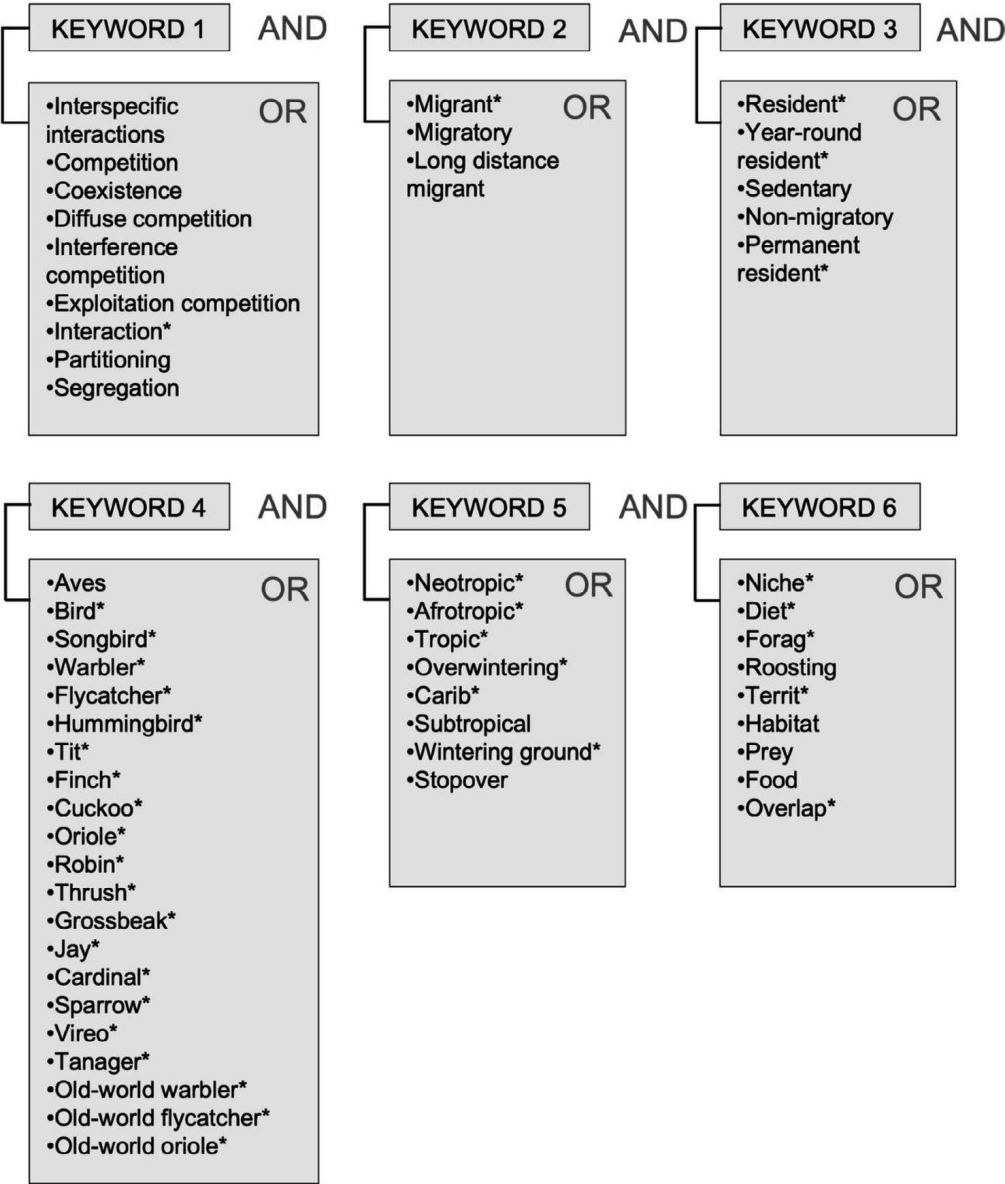
Keywords for scoping search using Boolean operators. Keywords were searched using the AND operator. The terms within each Keyword were searched using the OR operator.

We conducted a comprehensive search across Scopus and Web of Science, focusing on scientific publications regarding interspecific interactions between residents and migrants, specifically emphasizing evidence of migrants affecting residents in the tropics. Our search utilized 54 terms related to the main question within six keywords (Figure 1). We considered studies published in English, Spanish, and German between 1950 and August 2024. For the search in Scopus, we selected the option to search in “All‐fields”. For Web of Science, we selected to search in title, abstract, and keywords since the *All‐fields* option would include references and other irrelevant secondary information, exaggerating the results.

### 2.2 Screening Phases and Synthesis

In the search, we obtained five hits from Scopus and 103 hits from Web of Science. The results underwent a two‐phase screening process: the first involved examining titles, keywords, and abstracts, while the second examined the full text. From the first screening, 35 papers directly or indirectly addressing the main questions were selected for further reading. We retained papers from scientific journals (28), conference proceedings (4), and unpublished theses (3). The preselected papers were read and filtered for content and pertinence. We excluded studies on the impact of residents on migrants without resident ecology details, studies on migrant‐migrant interactions, competition between migratory and sedentary subspecies, and migrant‐resident interactions during the breeding season.

Fifteen papers presented relevant data on territoriality, aggression, diet, foraging niche, or habitat use involving at least one resident‐migrant species pair in the tropics. These papers comprised observational and experimental studies conducted in America and Africa. No studies were identified from Asia or featuring Austral, partial, or intratropical migrants in non‐breeding grounds. While Cornwallis (1975) provides a comparative ecological study of eleven species of wheatears (Oenanthe genus) in southwest Iran, most of the species examined are breeders in Iran, with only four species exhibiting short‐distance migration within Iran, resulting in neighboring breeding and wintering areas. Furthermore, this study relies on casual rather than systematic foraging observations, does not differentiate between behavior or diet during breeding and non‐breeding seasons, and does not investigate agonistic interactions on wintering grounds. As such, it falls outside the scope of this review and was not included.

Finally, we looked at the references and citations of these 16 papers using Web of Science and Litmaps. Litmaps is a citation network tool powered by AI that allows researchers to track, visualize, and explore connections between scientific publications over time. Using both tools ensured our review was thorough. This process contributed three more studies, bringing the total to 19.

We summarized the findings in two tables. Table 1 groups the studies by the strength of evidence for interspecific competition, aggression, territoriality, habitat shifts, and diet overlap. Table 2 provides details on each study, such as location, interacting species, effort, and other relevant information. Scientific names are not included in the text, as they can be found in Table 2. All reported results are statistically significant unless explicitly stated otherwise. Throughout the text, it will be indicated whether any of the necessary or sufficient conditions for the existence of interspecific competition are met by using its abbreviation (e.g., (NC1) necessary condition 1) [explanation below].

**TABLE 1:** Summary of evidence strength for interspecific competition conditions and behavioral/niche criteria based on Dhondt (2012).

Criteria	Strong evidence	Weak evidence	No evidence	Not evaluated
SC1	1, 4, 5, 6, 7, 8, 10, 15, 16, 19	2, 3, 9, 11, 12, 14, 17		13, 18
Intraspecific territoriality	4, 5, 7, 8, 10, 11, 12, 14, 16, 17, 19	1		2, 3, 6, 9, 13, 15, 18
Interspecific territoriality	1^a^, 4, 5, 6, 10, 11, 12, 16, 19	17	14	1, 2, 3, 7, 8, 9, 13, 15, 18
Intraspecific aggression	1, 2, 4, 5, 8, 10, 11, 12, 14, 16, 19	17	7	3, 6, 9, 13, 15, 18
Interspecific aggression	1, 2, 4, 5, 6, 8, 10, 11, 12, 14, 16, 19	2, 13, 17	3, 7	9, 15, 18
Foraging niche overlap	3, 5, 8, 9, 10, 13, 14, 15, 16, 17, 18	1, 11, 12, 19		2, 4, 6, 7
Foraging niche shifts/segregation	1, 2, 3, 5, **7**, **11**, 12, **13**, 18		**14**	4, 6, 8, 9, 10, 15, 16, 17, 19
Diet overlap	8, 15	5, 6		1, 2, 3, 4, 7, 9, 10, 11, 12, 13, 14, 16, 17, 18, 19
Habitat overlap	1, 3, 8, 10, 11, 12, 15, 16, 17, 19	5		2, 4, 6, 7, 9, 13, 14, 18
Habitat use shift/segregation	3, 11, 14		17	1, 2, 4, 5, 6, 7, 9, 10, 12, 13, 15, 16, 18, 19
NC1	3, 4, 5, 6, 7, 8, 10, 11, 15, 16, 17, 18, 19	1, 2, 9, 12, 13, 14		
NC2	1, 2, 4, 5, 7, 8, 10, 11, 12, 14, 15, 16, 17, 19			3, 6, 9, 13, 18
NC3	1, 4, 5, 6, 8, 9, 10, 14, 15, 16, 17, 19	2, 3, 7, 11, 12, 13, 18		

*Note:* The numbers correspond to specific studies listed in Table 2. Evidence is categorized as strong, weak, no evidence, or not evaluated for each criterion. Foraging niche and habitat use: Shift: not bolded, Segregation: underlined, Shift and segregation: Bolded.

^a^ The resident defends their territories interspecifically, but the migrants do not establish territories in Zimbabwe.

**TABLE 2:** Studies included in this review.

Location/ecosystem	Study period	Interacting species		Effort (years)	References	Study id.
		migrant	resident			
Zimbabwe. Deciduous broad‐leaved woodland	February–March	Eurasian Golden Oriole	African Golden Oriole African Black‐headed Oriole	1	Baumann (2001)	1
Popayán, Colombia. Tropical Cloud Forest	Year‐round	19 migratory warblers	Hepatic Tanager Golden Tanager Dusky‐capped Flycatcher Slate‐throated Redstart Brown‐capped Vireo Tropical Parula 11 residents	1	Chipley (1976)^a^	2
Ghana. Savanna	January–March November–May	Willow Warbler Pied Flycatcher Melodious Warbler	Northern Crombec Rufous Cisticola Senegal Eremomela	2	Gbemiga (2014)^a^	3
Chiapas, Mexico. Tropical moist forest	September–April	Yellow Warbler 14 migrants	12 residents	1	**Greenberg and Salgado Ortiz (** **1994** **)** ^a^	4
Yucatán, Mexico. Tropical Dry Forest	October–March	White‐eyed Vireo	Mangrove Vireo	3	Greenberg, Macias Caballero, and Bichier (1993) and Greenberg et al. (1993)	5
Chiapas, Mexico. Oak forest	January–March	Yellow‐rumped Warbler	White‐eared Hummingbird	2	Greenberg et al. (1993)^a^	6
Chiapas, Mexico. Shade‐grown polyculture organic coffee	June–July December–January	Multiple species	Rufous‐capped Warbler	1	**Jedlicka et al.** **(** **2006** **)**	7
Jamaica. Coastal mangrove forest	January–March	American Redstart	Yellow Warbler	1	Kent et al. (2022)	8
Gamboa, Panamá. Tropical Humid Forest	September–May	7 migrants	23 insectivorous residents	2	Poulin and Lefebvre (1996)^a^	9
Jamaica. Coastal mangrove forest	January–April	American Redstart	Yellow Warbler	3	Powell et al. (2021)	10
Kenya. Savanna	August–December	Willow Warbler	Green‐backed Camaroptera Yellow‐breasted Apalis Tawny‐flanked Prinia	1	**Rabøl** **(** **1987** **)** ^a^	11
Kenya. Savanna	August–November	Willow Warbler	Green‐backed Camaroptera Yellow‐breasted Apalis	1	**Rabøl** **(** **1993** **)** ^a^	12
Zimbabwe. Woodland	December–February	Willow Warbler	Burnt‐necked Eremomela	1	Salewski, Jones, and Vickery (2002)	13
Ivory Coast. Savanna	September–April	Pied Flycatcher	Gambaga Flycatcher Senegal Batis Red‐bellied Paradise Flycatcher Brown‐throated Wattle‐eye	1	Salewski, Bairlein, and Leisler (2003)^a^	14
Jamaica. Coastal mangrove forest and scrub	January–March	American Redstart	Yellow Warbler	1	Southwell (2018)	15
Puerto Rico. Second growth deciduous forest	September–April	Cape May Warbler Northern Parula Prairie Warbler	Adelaide's Warbler	4	Staicer (1992)	16
Puerto Rico. Dry Forest	March–April	American Redstart	Adelaide's Warbler	3	Toms (2011, 2013)	17
Campeche, Mexico. Tropical Dry Forest	June–September September–February	19 migrants	Great Kiskadee Lesser Greenlet White‐bellied Wren Gray‐throated Chat 16 residents	2	**Waide (** 1981)^a^	18
Sahel area in Africa. Savanna	October–March	17 migrants	Spectacled Warbler 10 residents	8	Zwarts, Bijlsma, and Van der Kamp (2023)^a^	19

*Note:* Details of the location and ecosystem, study period, interacting species (migrants and residents), effort in years, and corresponding references. Each study is assigned an ID number for cross‐referencing with Table 1. Bolded references indicate studies that quantified arthropod abundance and/or change in time. Scientific names: ADWA: *Setophaga adalaidae*, AGOR: *Oriolus auratus* , AMRE: *Setophaga ruticilla* , ANEU: *Euphonia musica* , BANA: *Coereba flaveola* , ABHO: *Oriolus larvatus* , BCVI: *Vireo leucophrys* , BTWE: *Platysteira cyanea* , BNER: *Eremomela usticollis* , CMWA: *Setophaga tigrina* , CCWA: *Oreothlypis superciliosa* , DCFL: *Myiarchus tuberculifer* , EGOR: *Oriolus oriolus* , GAFL: *Muscicapa gambagae* , GOTA: *Tangara arthus* , GTCH: *Granatellus sallaei* , GKIS: *Pitangus sulphuratus* , GBCM: *Camaroptera brachyura* , HETA: *Piranga flava* , LEGR: *Pachysilvia decurtata*, MAVI: *Vireo pallens* , MEWA: *Hippolais polyglotta* , NOCR: *Sylvietta brachyura* , NOPA: *Setophaga americana* , PARE: *Myioborus pictus* , PIFL: *Ficedula hypoleuca* , PRAW: *Setophaga discolor* , RBPF: *Terpsiphone rufiventer* , RUCI: *Cisticola rufus* , RCWA: *Basileuterus rufifrons* , SEBA: *Batis senegalensis* , SEER: *Eremomela pusilla* , STRE: *Myioborus miniatus* , SPWA: *Curruca conspicillata*, TFPR: *Prinia flavida*, TRPA: *Setophaga pitiayumi* , WBWR: *Uropsila leucogastra* , WEHU: *Hylocharis leucotis* , WEVI: *Vireo griseus* , WIWA: *Phylloscopus trochilus* , YEWA: *Setophaga petechia* , YBAP: *Apalis flavida* , YRWA: *Setophaga coronata* .

^a^ Studies done at the community level. Studies without a symbol studied 1–3 species.

### 2.3 Assessment of the Evidence for Interspecific Competition

We assessed the evidence for interspecific competition for each paper based on Dhondt's (2012) recommended criteria. These criteria include three *necessary conditions* that need to be documented for interspecific competition to be possible and three *sufficient conditions*, each proving the existence of interspecific competition. The three necessary conditions (NC) are: (NC1) One or more resources are limited; (NC2) Intraspecific competition occurs within populations of interacting species; and (NC3) Interacting species overlap in the use of these specific limited resources. The sufficient conditions (SC) are: (SC1) resource use by one species affects the resource availability or use by another, (SC2) fitness of one of the interacting species is reduced by the presence of the other species, (SC3) distribution or abundance of at least one of the interacting species is reduced by the presence of another (Dhondt 2012). Given that we did not find any long‐term study of a resident tropical species in which both adult survival and reproductive success were measured in relation to the abundance of overwintering migrants, we also include in our review studies describing behavioral interactions that should lead to a negative effect on the residents' fitness. We assessed the strength of the evidence for each criterion in each study. A criterion was classified as “strong evidence” if the authors explicitly or implicitly indicated it was met. “Weak evidence” was assigned when data suggested the criterion might be met but with insufficient support. The term “not demonstrated” was used for criteria evaluated in the original study, but for which no evidence was found. Finally, we noted when a criterion was “not evaluated” in the study.

## 3 Results

Our review revealed that migrants and residents undergo competition in the tropics. Ten studies provide strong evidence of migrants' impact on residents (SC1 in Table 1). In Table 1, we summarized the strength of evidence (strong, weak, not demonstrated, not evaluated) for various criteria related to interspecific competition across 19 studies. First, we classified studies documenting any of the sufficient conditions, as these provide evidence of interspecific competition whereby migrants impact residents. No studies assessed or contained data to evaluate either SC2 or SC3. Next, we listed studies in which interspecific territoriality or aggression had been described since these are mechanisms for interference competition (Dhondt 2012; Drury, Cowen, and Grether 2020; Freeman 2019). Following this, we listed the studies that assessed changes in foraging niche, diet overlap, habitat overlap, and habitat use. Finally, due to the small number of studies that provided convincing evidence for the existence of an effect of migrants on residents, we also included studies that provided data supporting any of the three necessary conditions. These studies were included because additional research in these systems has the potential to test the extent to which interspecific competition could occur. Broadly, the review presents evidence of territoriality and aggression, shifts in foraging niches, and diet overlap between migrants and residents.

### 3.1 Interspecific Territoriality and Aggression

Although interspecific territoriality is not strictly a sufficient condition to prove the existence of interspecific competition, as we cannot conclude that resident fitness would be impacted, it certainly reduces resource availability and access for the residents. When migrants establish themselves, they exclude residents from such territories and the resources within them. Additionally, the territorial defense can decrease foraging and resting time and possibly increase predation (Davies and Houston 1981; Toms 2011), which could impact fitness.

In most studies, migrant and resident birds exhibited high habitat overlap during the wintering period, with interspecific territoriality frequently observed in both groups (Chipley 1976; Gbemiga 2014; Greenberg, Macias Caballero, and Bichier 1993; Greenberg et al. 1993; Powell et al. 2021; Rabøl 1993; Staicer 1992; Zwarts, Bijlsma, and Van der Kamp 2023). This overlap was evident in five studies in the Americas, where interspecific territoriality was confirmed among warblers (Greenberg et al. 1993; Greenberg, Salgado Ortiz, and Caballero 1994; Powell et al. 2021; Staicer 1992; Toms 2013), and vireos (Greenberg, Macias Caballero, and Bichier 1993; Greenberg et al. 1993). Two notable studies conducted in Puerto Rico with color‐banded warblers are those by Staicer (1992) and Toms (2011, 2013). In a comprehensive four‐year research Staicer examined space use and site fidelity of migrants. Although the study focused on three overwintering warblers (Cape May, Northern Parula, and Prairie), it also provided detailed information on the interactions with the resident Adelaide's Warbler. The findings revealed significant interspecific territoriality (SC1) among all species except the Prairie Warbler. This behavior was characterized by frequent aggressive interactions both within (NC2) and between species, suggesting food limitation (NC1). Overall, 10% of the observations were aggressions, chases, and oustings (0.97 aggressive interactions/h), with interspecific interactions more common than intraspecific interactions (NC3).

With some similarities, Toms' study (Toms 2011, 2013) looked at the foraging niches and responses to playback and decoys of the resident Adelaide Warbler and the American Redstart, finding a high degree of overlap in foraging location and attack technique, along with aggressive responses to both conspecific and heterospecific playback trials. The residents displayed marked territoriality, responding more strongly to intraspecific than interspecific trials and exhibiting less territory overlap with conspecifics (NC2, ~NC1). This pattern suggests that intraspecific competition among resident individuals plays a more significant role than interspecific competition with American Redstarts. Additionally, vocal interactions between them were common throughout the wintering period, and Redstarts were commonly observed using aggressive body posturing in encounters with the residents. Although not quantified, physical attacks between migrants and residents were frequently observed, with both species equally initiating the attacks early in the season while mostly initiated by the residents later in the season. A limitation of the study—using only one playback song and one decoy—renders the evidence somewhat inconclusive. Nonetheless, as the necessary conditions (NC1, NC2, NC3) are met and there is some indication of frequent interspecific territoriality and aggression, competition between these species is likely, especially in areas with higher densities of Adelaide Warblers, as suggested by the author. These two studies highlight the complexity of the dynamics at play in warbler communities, where competition for resources has been observed (Sherry and Kent 2022).

In multiple studies using playback experiments or systematic observations, aggression was the mechanism through which migrants limited the access to resources for the residents. An 8‐year study conducted by Zwarts, Bijlsma, and Van der Kamp (2023) in the subtropical Sahel region of Africa found frequent interspecific aggression within (NC2) and among migrants and between migrants and residents (SC1). Our analysis of the results in table 1 of Zwarts, Bijlsma, and Van der Kamp (2023) showed that migrants exhibited aggression toward resident species in 11% of the observations. During an average observation period of 113 min across six migratory species, there were 0.89 aggressions toward residents per hour of observation, with values ranging from 0.2 to 2.0 (calculations derived from supplementary information data in Zwarts, Bijlsma, and Van der Kamp 2023). Baumann (2001) studied the wintering behaviors of closely related orioles in Zimbabwe. Although the migrants do not hold territories, Baumann found that 48% of the interactions between the resident African Golden Oriole and the migrant Eurasian Golden Oriole were aggressions (NC1, NC3). Playback experiments confirmed the strong responses of both species to conspecific (NC2) and heterospecific songs, with stronger responses in the residents. He also noted an incomplete niche segregation, with residents foraging at lower heights, internal branches, and smaller trees than migrants. It is essential to highlight that Baumann's fieldwork occurred in February–March in southern Africa at the end of the residents' breeding season (August to January) (Walther and Jones 2020). Thus, it is possible that the high rate of aggression was at least partially due to an overlap with the end of the breeding period.

Similarly, in the Americas, three studies with color‐banded individuals found interspecific territoriality to be common, with frequent aggressions over specific limited food resources (NC1), such as standing trees within pastures (Greenberg and Salgado Ortiz 1994), fruiting trees (Greenberg, Macias Caballero, and Bichier 1993; Greenberg et al. 1993), or trees infested with homopterans (Greenberg, Macias Caballero, and Bichier 1993; Greenberg et al. 1993). The Yellow Warbler, White‐eyed Vireo, and Yellow‐rumped Warbler were highly territorial at these limited resources, respectively, keeping residents from accessing them (NC3, SC1). In focal observations, the Yellow Warbler directed 25% of chasings to foliage‐gleaning insectivorous residents (NC3, SC1), with most species attacked an average of 0.5 times per hour, and 11% of the attacks were directed to conspecifics (NC1 & 2) (Greenberg and Salgado Ortiz 1994). Additionally, the authors observed 314 events in which the Yellow Warblers chased other birds, including the Lesser Greenlet (4%), Paltry Tyrannulet (3%), White‐collared Seedeater (2%), White‐bellied Emerald, and Green‐breasted Mango (< 1%), among other resident species (1%–2%).

The resident Mangrove Vireo was chased away by the territorial migrant White‐eyed Vireo in 45% of their visits to fruiting trees and overall tended to spend less time in these trees when the migrants were present (NC1, SC1) (Greenberg, Macias Caballero, and Bichier 1993; Greenberg et al. 1993). Playback experiments with calls showed that 70% of the trials of migrants' “chatter calls” on residents resulted in the residents responding aggressively by approaching less than 5 m to the speaker and with flyovers (20%). At Cerro Huitepec, Mexico, Yellow‐rumped Warblers showed high territoriality and overt aggression against other migrants, conspecifics, and residents (NC1 & 2) at patchy trees infested with scale insects (NC3, SC1) that produced honeydew. While most aggressions were directed at migrants, some residents were also attacked, such as the White‐eared Hummingbird (5%), Crescent‐chested Warbler, Painted Redstart, and Slate‐throated Redstart (2.6%–3%) (Greenberg, Macias Caballero, and Bichier 1993; Greenberg et al. 1993).

Consisting with these findings, Powell et al. (2021) provided compelling evidence of intraspecific and interspecific competition between the migratory American Redstart and the resident Yellow Warbler. Although their emphasis was primarily on the American Redstart's wintering ecology, their findings extended to Yellow Warblers exhibiting overt and frequent territoriality and aggression toward male Redstarts (176 aggressive encounters recorded across three winters) (NC1 & 2). Migrants occupied territories in close proximity to the residents, with an average of six male Redstarts' territories overlapping a Yellow Warbler pair territory (NC3). Female Redstarts were excluded from high‐quality habitats (mangroves) (NC2). Removal experiments revealed territory as a limited resource (NC1), as neighboring Redstarts' yearlings took over when pairs of Yellow Warblers were temporarily removed (SC1). Unfortunately, the removal experiment was not conducted in the reverse direction, leaving us uncertain of whether the competition was reciprocal, although it is expected to be the case (L. Powell *pers. comm*.).

Collectively, these studies showed that aggressive responses to heterospecifics were common between migrants and residents in different habitats of Africa and the Americas. Residents faced territory reduction, restricted access to food sources, and devoted energy to sustained vigilance and aggression.

### 3.2 Foraging Niche Shifts

Studies in which the residents' foraging behavior and microhabitat use changed when migrants arrived suggest the possibility of an impact on residents. Those cases in which such a shift reverted when migrants departed would provide strong evidence that the migrants negatively impacted residents.

Among the studies investigating foraging niche dynamics, many found resident species altering their foraging heights when migrants were present (Baumann 2001; Chipley 1976; Gbemiga 2014; Jedlicka et al. 2006; Rabøl 1987; Waide 1981), mainly finding that resident species shifted to lower foraging heights. Fewer studies, however, have also explored foraging behavior beyond the period of co‐occurrence, which could be instrumental in identifying niche shifts. These include the studies by Chipley (1976) (year‐round), Gbemiga (2014) (co‐occurrence and the period after migrants departure), Jedlicka et al. (2006), and Waide (1981) (co‐occurrence and post‐breeding peak periods). All four studies provided evidence of residents (Chipley *n* = 6, Gbemiga *n* = 3, Jedlicka *n* = 1, Waide *n* = 7) shifting their foraging height to lower branches when the migrants were present. This change was abruptly reversed upon migrants' departure in Gbemiga's and Chipley's studies.

In a shade‐grown polyculture of organic coffee system in Mexico, Jedlicka et al. (2006) found that despite consistent arthropod abundance in the canopy and understory across seasons, the resident Rufous‐capped Warbler foraged 27% more frequently in the understory when the migrants were present (Jedlicka et al. 2006). This shift coincided with a 58% decrease in large arthropods (> 5 mm) availability in control branches (NC1), highlighting the role of predation on the availability of large prey in this stratum. The decline was attributed to the influx of migratory species in the wintering period, which decreased the availability of large arthropods (NC3) and triggered the observed shift to the understory. This conclusion was supported by the assessment of foraging success rates of Rufous‐capped Warblers across seasons and strata, revealing that the resident had a higher success rate in the canopy compared to the understory. Additionally, the authors observed intraspecific territoriality (NC2) among residents, but no aggressive interactions were noted between migrants and residents. This suggests that aggression was not the driving mechanism behind the observed shift. The authors proposed alternative explanations, including the collective effects of exploitation competition and the Breeding Demand hypothesis (differential dietary preferences of adults between life cycles) (Skutch 1950). This result is also consistent with the hypothesis of diffuse exploitation competition by Sherry and Kent (2022), requiring further studies. In summary, the Rufous‐capped Warbler was impacted by the presence of migrants having limited access to large arthropods in the canopy and lower foraging success (SC1), which could influence their fitness.

Gbemiga's study in northern Ghana also revealed that resident birds altered their foraging height when migrants were present. Residents shifted to lower foraging heights, narrowing their vertical range. This led to an increased niche overlap among resident species (Morisita Horn index 0.87–0.97), potentially intensifying competition. As migrants departed, residents expanded their foraging height range, reducing niche overlap with the other resident species (average Morisita Horn index 0.45). Also, within same foraging guild pairs of migrants and residents, there was a moderate to high niche overlap (Morisita Horn index 0.42–0.98) when assessing foraging technique, height, and habitat type (NC3). This overlap indicates their ecological similarity and may help explain the shift in foraging height. The fact that residents' foraging ecology changed when migrants were present and the reversed niche overlap suggests the possibility of a competitive release, consistent with the Niche Variation Hypothesis. This hypothesis states that when a population no longer faces competition from another species' individuals, the niches will expand because either all individuals will start using a wider variety of resources or the individuals will increase inter‐individual resource use (Van Valen 1965).

Chipley's study (1976) in Colombia showed that as the migrants arrived, six residents shifted to lower foraging heights, whether the birds foraged solitarily or in mixed flocks with migrants (NC3). Chipley observed that 15% of all agonistic behaviors were interspecific interactions (SC1). In October, the resident Tropical Parula which changed its foraging height, also showed a surge in intraspecific aggression (NC2), which coincided with the peak arrival and abundance of migratory passerines. As in Gbemiga's study, this pattern suggests a potential escalation in intraspecific competition among residents during periods of higher influx of migrants.

In Mexico, Waide (1981) found that two resident species changed their foraging technique and substrate in addition to the shifts in foraging height. Moreover, the foraging height shift in the resident Gray‐throated Chat led to a significant reduction in niche overlap with four migratory and one resident species. Although Waide's conclusions did not support resource competition, we suggest that these significant shifts indicate either a way to mitigate competition or a consequence of it.

Likewise, in Kenya, Rabøl's (1987, 1993) research on food availability and potential competition between migratory Willow Warblers and insectivorous residents revealed residents altering their foraging ecology (preferred tree height, foraging height, and distance to the trunk) and frequent interspecific aggression from the migrants (SC1). Such aggressions became more frequent toward the end of the study period (early northern winter) when the conditions were dryer (NC1). Similarly, Salewski, Jones, and Vickery (2002) found that in Zimbabwe, the residents Burnt‐necked and Green‐capped Eremomelas participating in mixed‐species flocks with migrants altered their foraging ecology by foraging more frequently in outer branches than they did when in monospecific flocks, although this result was only marginally significant (*p* = 0.06). Conversely, Salewski, Bairlein, and Leisler (2003) observed a high overlap (NC3) in foraging techniques, microhabitats, and substrates between most resident birds and the migrant Pied Flycatcher in Ivory Coast. While the residents did not exhibit any noticeable shifts, the migrant Pied Flycatcher spent more time in the lower stratum as conditions became drier. While not all the necessary conditions are met (or evaluated) in these studies, and intraspecific competition was only witnessed in Chipley's and Jedlicka's study, we consider that these studies provide some indications of changes in residents' foraging ecology and behavior coinciding with migrants' arrival and stay.

### 3.3 Diet Overlaps

When the necessary conditions are met, and competitor species exhibit significant overlap in their diets, it may indicate exploitative interspecific competition (SC1) (Dhondt 2012). Conversely, incomplete overlap and distinct foraging niches could mitigate such competition. Whereas some dietary studies have included resident bird species in the neotropics (Jedlicka et al. 2021; Poulin and Lefebvre 1996; Sherry 1984; Snow 1981), comprehensive knowledge of residents' diets across seasons remains limited. Nevertheless, five studies found dietary overlap between ecologically similar species of migrants and residents in the Neotropics.

Two studies developed in the same Jamaican mangrove investigated the interactions between the resident Yellow Warbler and the migratory American Redstart, two species frequently regarded as competitors (Marra 2000). Using complementary methods, Southwell (2018) (metabarcoding of fecal samples) and Kent et al. (2022) (stomach regurgitations) found high dietary overlap (NC3, SC1) between these species at the order level. Both studies found that the overlap occurred at low‐nutritional value prey items (e.g., ants, termites, true bugs) whose availability is supposed to be constant year‐round (Johnson et al. 2005), instead of on high‐nutritional value prey (e.g., caterpillars, beetles' larvae, spiders), as would be expected. These results are consistent with Greenberg's breeding currency hypothesis (1995) as tested by Johnson et al. (2005). Kent et al. (2022) did not find evidence of dietary specialization. In contrast, Southwell (2018) identified that residents predominantly preyed on caterpillars (Lepidoptera), while migrants predated more on flies (Diptera) and an order of insects (Strepsiptera) exclusively found in the resident (note that this order consists of endoparasites, which are often easy to miss). Molecular analysis of Lepidoptera and Diptera OTUs (Operational Taxonomic Units) further revealed unique prey occurrences for each species (Southwell 2018), which indicates partitioning, possibly due to the high dietary overlap. We hypothesize that perhaps Kent et al. (2022) were unable to observe such partitioning due to limitations of their study, such as the sample size discrepancies (Redstarts = 5, Yellow Warblers = 17), the degradation of prey through digestion and the low taxonomic resolution of prey, which may have obscured prey specialization and influenced the assessment of diet overlap. Nonetheless, the consistent pattern of dietary overlap in two independent studies, along with the overt and frequent aggression observed by Powell et al. (2021) and many others, reinforces the notion of limited resources and the possibility of exploitation and interference competition.

By collecting 1627 stomach content samples of insectivorous birds in Panama, Poulin and Lefebvre (1996) examined dietary “preferences” and overlap, finding high dietary similarity within migratory species, low similarity within resident species, and minimal similarities between the overall diets of residents and migrants. Nonetheless, there was a high dietary similarity between four migratory and five resident species of foliage‐gleaning, aerial‐foraging, and ground‐foraging insectivore guilds. This high dietary similarity between some species suggests the possibility of exploitation competition, which could be confirmed with further studies (Sherry and Kent 2022).

## 4 Discussion

Although it remains a notably underdeveloped topic within the study of competition, this review uncovered widespread evidence of migrants and residents interacting during the overwintering period. These interactions include interspecific territorial defense, agonistic behaviors, shifts in residents' foraging behavior and microhabitat use that coincides with the arrival and stay of migrants, and high dietary overlap between same‐guild migrants and residents. Despite this evidence, studies focusing on how migrants may impact resident populations are limited, especially compared to the relatively common exploration of residents' impacts on migrants. This disparity may stem from early assumptions that migratory species had a negligible influence on residents (Howell 1971; Leisler 1992; Ricklefs 1992; Salewski, Jones, and Vickery 2002; Willis 1966), along with more recent attention on the ecology of migrants throughout their life cycles, driven by their acute population declines (Rosenberg et al. 2019; Sanderson et al. 2006). Additionally, the limited observation of agonistic encounters has often been interpreted as a lack of competition. However, Zwarts, Bijlsma, and Van der Kamp (2023) argue that competition varies significantly by habitat type and bird density. In savannah trees (e.g., in the Sahel), high bird densities result in frequent agonistic interactions, reflecting intense competition. In contrast, in woody savannahs and forests further south in Africa, where bird densities can be lower, encounters between individuals are rare, leading to fewer observed agonistic interactions. Similarly, Rabøl (1987, 1993) observed more frequent agonistic interactions during years with higher migrant densities (1983) compared to years with lower densities (1993), as discussed in Section 4.4. Moreover, competition may still occur in less direct ways (e.g., through resource depletion) (Jedlicka et al. 2006; Sherry and Kent 2022). Practical and funding challenges in tropical regions also restrict research into these dynamics, as most funding is directed to applied sciences.

Interestingly, we observed distinct approaches to studying interspecific competition between the Palearctic‐Afrotropical and Nearctic‐Neotropical systems. African studies primarily focused on foraging ecology and habitat use. In contrast, American studies focused on diet overlap, territoriality, and foraging niches, possibly influenced by MacArthur's work (MacArthur 1958). The presence of competitive interactions across both systems suggests a generalized pattern and indicates avenues for further investigation. Applying Dhondt's (2012) criteria to assess interspecific competition offers a valuable framework for distinguishing between the potential (Necessary Conditions) and actual occurrence (Sufficient Conditions) of competition, enhancing our understanding of these interactions.

### 4.1 Agonistic Interactions and Playback Experiments

Some studies noted that while territorial behavior was common, aggressive interactions between different species were rare, leading some researchers to conclude that competition was not occurring. However, a lack of frequent aggressive encounters does not rule out competition as it could depend on the species' densities and habitat type (Zwarts, Bijlsma, and Van der Kamp 2023). Rigorous evidence of interspecific territoriality (e.g., using color‐banded individuals), as highlighted by Dhondt (2012) and Drury, Cowen, and Grether (2020), denotes ongoing interspecific competition. Freeman (2019) supports this idea, arguing that aggressive behavior between species is a reliable sign of competition. This suggests that instances where migrants and residents defended territories interspecifically, as presented in Section 3.1, provide support for SC1.

The use and defense of territories by individuals of one species affects the use by individuals from another species, indicating interference competition. Most such interactions occur between closely related species, except for the resident White‐eared Hummingbird and the migrant Yellow‐rumped Warbler (Greenberg, Macias Caballero, and Bichier 1993), and the White‐bellied Emeralds and Green‐breasted Mangoes attacked by Yellow Warblers (Greenberg and Salgado Ortiz 1994). These are the only cases in which we found aggression between different‐order species.

Frequent reports on the scarcity of agonistic encounters in the field should be carefully interpreted, given the challenges of detecting and documenting agonistic interactions with most current field methods (Salewski and Jones 2006). Only a few studies, like those by Staicer (1992), Greenberg, Macias Caballero, and Bichier (1993), Greenberg et al. (1993), Greenberg and Salgado Ortiz (1994), Salewski, Almasi, and Schlageter 2006, and Zwarts, Bijlsma, and Van der Kamp (2023), have provided quantitative data on aggression, including metrics such as the rate of aggressions per hour or the ratio of agonistic to non‐agonistic interactions. The prevailing tendency among authors to rely on anecdotal estimations (e.g., few, rare, uncommon) rather than precise quantitative data limits our ability to determine the frequency of agonistic behaviors. This distinction is essential because aggression itself is not evidence of competition. We recommend that future studies maintain detailed quantitative records for a more comprehensive understanding of these interactions.

A related challenge is the need for consistent methods (Kroodsma et al. 2001) and ways of measuring response levels when using playback experiments to test intra and interspecific competition. Many studies using playback used subjective behavioral scales to determine if the response was or was not indicative of competition. For some authors, strong responses must include active singing or calling (like they often do in breeding grounds); for others, approaching the speaker or flying over was enough. Assumptions regarding breeding ground behavior can harm experiments conducted in non‐breeding habitats: first, because species behavioral responses may vary between and within species; second, because responses can vary by sex, age, and social hierarchy (e.g., yearlings vs. adults, floaters vs. territory owners); and third, because of neighbor‐stranger discrimination effects (Lovell and Lein 2004), which may be predisposed by the source of the recording used (Kroodsma 1990). Thus, selecting suitable recordings (Kroodsma et al. 2001) and establishing clear criteria to interpret playback trials is necessary for a more objective assessment of competition across systems.

### 4.2 Residents' Niche Shifts

The behavioral adjustment of residents shifting to lower foraging heights observed across various ecosystems in the old and new world suggests potential microhabitat displacement, aligning with Jedlicka et al.'s (2006) prediction of its widespread adaptive nature. Nonetheless, whether the height shifts indicate that the migrants displaced residents, or whether the shift obeys seasonal changes or residents tracking a particular prey in the understory remains unclear. Either way, the shift could alleviate competition with migrants (Waide 1981), but it could also increase competition with conspecifics or other resident species (Chipley 1976; Gbemiga 2014). Gbemiga's findings on residents' increased niche overlap in the dry season and Chipley's findings on residents' higher intraspecific aggression correlated with migrants' abundance imply that migrants could potentially impact residents not only through exploitation and interference but also indirectly by intensifying residents' intraspecific competition. To our knowledge, this has not been described elsewhere.

Could shifts in foraging height influence survival and reproductive fitness? It is known that competition on nonbreeding grounds carries over to the survival and reproductive success of migratory species (Dossman, Matthews, and Rodewald 2018; Norris et al. 2004; Powell et al. 2021; Reudink et al. 2009) Surprisingly little attention has been paid to assessing the impact on resident species when the migrants are present or once they are gone. Although some studies on the survival and fitness of tropical bird species have been conducted (Blake and Loiselle 2008; Johnston et al. 1997; Karr, Nichols, and Klimkiewicz 1990), none have addressed the possible carryover effect of interspecific competition with migratory species during the non‐breeding season on the residents' reproductive success. On the other hand, shifts in foraging substrate, height, or distance to the trunk appear to be common among competitors when in sympatry (Alatalo and Moreno 1987; Hogstad 1978), as shown in some studies in this review. Such a shift might impact (1) the access and quality of prey and (2) how detectable individuals are to predators (Jedlicka et al. 2006). Foraging on outer branches could imply that the prey encountered might be different in composition, abundance, and nutritional value from that of the inner branches (Kent, Peele, and Sherry 2019). Additionally, birds foraging in the periphery would be more exposed to avian predators, which could ultimately influence survival.

### 4.3 Bias in Dietary Overlap Studies

Our review revealed a clear bias in dietary studies: they all focused on birds that eat insects, ignoring other feeding guilds. This bias may arise from the difficulty associated with identifying plant materials in bird diets that can be present in some “insectivorous” birds, such as warblers (T. Sherry *pers. comm*.). Although not yet fully understood, migrants consumption of fruits and seeds could potentially intensify competition with certain resident species—such as small flycatchers (e.g., *Tolmomyias*, *Mionectes*), tanagers, and some warblers—that rely to some extent on these food sources. Additionally, some migrants, such as the Cape May Warbler have been reported drinking nectar on their wintering grounds (Greenberg, Salgado Ortiz, and Caballero 1994; Latta and Faaborg 2002; Staicer 1992). This adds another layer of complexity to dietary studies due to the rapid digestion of nectar, which raises challenges for detection and identification with current methods.

Another issue is that most studies lacked taxonomic resolution in prey identification, which further complicates the estimation of dietary overlap (Kent et al. 2022; Poulin and Lefebvre 1996; Southwell 2018). Enhancing the resolution of dietary studies is essential to identify critical dietary items, allowing for a more accurate assessment of dietary overlap and the role of specific items in mitigating or exacerbating interspecific competition (Kent et al. 2022).

Complementary methods such as DNA metabarcoding of fecal samples and stable isotope analysis can be useful. Both methods provide insights into food assimilation over different timescales depending on the sampled tissue (Faria, Albertoni, and Bugoni 2018; Fox and Bearhop 2008; Herrera et al. 2003; Hoenig et al. 2022; Southwell 2018), making more evident the overlap and potential competition between migrants and residents. However, some shortcomings of metabarcoding are that it can only provide presence/absence data, since readings cannot be used to calculate the frequency of use, and it cannot discriminate between the different prey' life stages or tissue consumed (e.g., adult vs. larval, fruits vs. leaves) (Valentini, Pompanon, and Taberlet 2009). In contrast, although labor‐intensive, morphological approaches such as stomach regurgitations can provide this information but are limited in prey identification and accuracy due to prey degradation (Kent and Sherry 2020).

### 4.4 The Importance of Long‐Term Studies

One difficulty when studying tropical systems is the varying climate, which influences survival and reproduction (Jiguet, Brotons, and Devictor 2011; Smart, Smith, and Riehl 2021) and arthropods' abundance/availability (Sherry et al. 2016; Waide 1981), within and between years (Jedlicka et al. 2021; Rabøl 1987, 1993; Waide 1981). Few studies sampled their system for two years or more (mean 3 years), and even fewer considered the variation this might cause to the analysis. Waide (1981) pointed out that the dryer year in his study period showed significant shifts in foraging niches for some species, which was not evident in the previous year. Rabøl (1987) found resource scarcity and more frequent aggressions at the beginning of the dry season in one year, but in another, arthropod abundance was relatively constant (Rabøl 1993), and aggressions were less common. A macro study confirmed the impact of annual variation on the diets of migrants and residents across different feeding guilds (Jedlicka et al. 2021). These findings highlight the need for long‐term studies to account for year‐to‐year variation as competition appears to manifest more prominently toward the end of the dry season or in years with reduced resource availability (likely in El Niño years (Sillett, Holmes, and Sherry 2000)). Without meticulous and replicated long‐term research, these competitive dynamics may go undetected (Dhondt 2023).

### 4.5 What Do We Know About the Interactions at Stopover Sites?

This review found few studies done at stopover sites, which suggest that even during a short stay, migrants might influence changes in residents' behavior. Bensusan, Shorrocks, and Hamer (2011) showed a relationship between daily changes in migrants' abundance and territorial displays (calls and frequent patrolling) in the resident Sardinian Warbler ( *Sylvia melanocephala* ). The Cyprus Wheatear's ( *Oenanthe cypriaca* ) foraging height was strongly correlated with migrants' abundance (Randler 2013), positioning itself between congeneric migrants and migratory shrikes and flycatchers (Randler, Pentzold, and Pentzold 2015). At a Saharan oasis, residents were frequently observed attacking migrants and rarely chased by them (Salewski et al. 2007). However, Zwarts, Bijlsma, and Van der Kamp (2023) argue that high levels of agonistic behavior observed in Salewski et al.'s study are not unique to stopover sites but are instead influenced by the habitat type and bird density. Their comparison of data from the Sahel (October–March) and the southern Sahara (March–May) shows that similar levels of aggression occur in both areas, where birds occupy habitats with scattered thorny trees and reach high densities. These findings suggest that aggression is driven more by habitat characteristics and bird density than by the transient nature of stopover sites.

These behavioral changes still suggest potential short‐term impacts of passing migrants on residents, warranting further investigation into their influence on the foraging niche and the potential for transient competition.

## 5 Conclusions

Our review revealed evidence of residents being impacted by migrants, but further research is needed to explore the potential carryover effects on residents' fitness (survival and reproduction) due to such competitive interactions, just as it has been demonstrated for some migratory counterparts (Powell et al. 2021). The complexity of these interactions and the challenges of separating the effects of seasonality and competition underscores the need for deeper investigation. Dhondt's criteria (2012) offer a valuable lens to evaluate interspecific competition. Future research should aim to identify and quantify limited resources and test intra‐ and interspecific competition in order to assess the potential impacts on fitness, distribution, or abundance for residents and migrants.

To advance this research, it is crucial to identify appropriate methods or proxies to improve our ability to detect competition dynamics more effectively. Playback experiments have proven helpful in testing both intra‐ and interspecific competition (Dhondt 2012; Freeman 2019), but careful experimental design is needed to avoid pseudo‐replication (McGregor 2000; Kroodsma et al. 2001). Food‐manipulation experiments targeting residents would also provide evidence of competitive dynamics and their impacts on fitness (Cooper et al. 2015). Kent, Peele, and Sherry (2019) introduced an affordable method for quantifying available arthropod composition and abundance, and there are multiple ways to evaluate survival, reproductive fitness (Dhondt 2023), and changes in species distribution.

Additionally, shifts in microhabitat or foraging height might be better indicators of niche partitioning than changes in foraging technique, as few studies reported changes in technique (Salewski 2000; Waide 1981; Zwarts, Bijlsma, and Van der Kamp 2023). Also, the substrate in which the species forages (e.g., leaves, trunk, twigs) is particularly useful to compare the foraging niche of sympatric species (Kent and Sherry 2020). Recording microhabitat shifts through detailed observations of substrate, plant height, and location in the tree (height and distance from trunk) could provide valuable insights to detect the effects of competitive interactions. Furthermore, dietary studies using morphological and molecular identification would be advised for a more refined assessment of diet overlap, along with measurements of body condition and overlap with energy‐demanding processes such as breeding and molting. These tools will provide a clearer picture of resource partitioning and competition.

Furthermore, conducting observations before and after the presence of migrants at wintering grounds can enhance our ability to detect shifts in resource availability and foraging niches, as demonstrated in Gbemiga (2014) and Chipley (1976). Long‐term studies of individually marked birds (e.g., color and metal banded) meticulously carried out and replicated are essential to fully capture these dynamics, considering the variability of interspecific competition across species and years. Based on the cited literature, we recommend that such studies span a minimum of three years to ensure robust and reliable insights.

These approaches will enhance our understanding of migrant‐resident competition in the tropics, especially given that climate change influences migration dates (Lawrence et al. 2022), fecundity, and survival (Sillett, Holmes, and Sherry 2000) and possibly alters the length of the migrants' overwintering period (Cheng et al. 2019; Gutiérrez‐Carrillo et al. 2024). Finally, exploring how these biotic interactions impact the fitness of resident species (Guillaumet and Russell 2022) is key to designing effective conservation programs that promote species' long‐term sustainability.

## Author Contributions

**Kimberly C. Navarro‐Velez:** conceptualization (equal), investigation (lead), methodology (lead), writing – original draft (lead), writing – review and editing (equal). **André A. Dhondt:** conceptualization (equal), supervision (lead), writing – review and editing (equal).

## Conflicts of Interest

The authors declare no conflicts of interest.

## Data Availability

The authors have nothing to report.
